# Pharmacological tools to mobilise mesenchymal stromal cells into the blood promote bone formation after surgery

**DOI:** 10.1038/s41536-020-0088-1

**Published:** 2020-02-21

**Authors:** Tariq G. Fellous, Andia N. Redpath, Mackenzie M. Fleischer, Sapan Gandhi, Samantha E. Hartner, Michael D. Newton, Moïra François, Suet-Ping Wong, Kate H. C. Gowers, Adam M. Fahs, Daniel R. Possley, Dominique Bonnet, Paula Urquhart, Anna Nicolaou, Kevin C. Baker, Sara M. Rankin

**Affiliations:** 1 Inflammation, Repair and Development Section, National Heart and Lung Institute, Imperial College, London, SW7 2AZ UK; 2 Department of Orthopaedic Surgery, Beaumont Health, Royal Oak, MI USA; 3 Faculté de Pharmacie, Laboratoire d’Innovation Thérapeutique, UMR7200, Centre National de la Recherche Scientifique/Université de Strasbourg, LabEx Medalis, Illkirch, France; 4 Faculty of Biology, Medicine and Health, Manchester Academic Health Science Centre, Laboratory for Lipidomics and Lipid Biology, Division of Pharmacy and Optometry, School of Heath Sciences, The University of Manchester, Manchester, M13 9PT UK

**Keywords:** Regeneration, Preclinical research, Mesenchymal stem cells

## Abstract

Therapeutic approaches requiring the intravenous injection of autologous or allogeneic mesenchymal stromal cells (MSCs) are currently being evaluated for treatment of a range of diseases, including orthopaedic injuries. An alternative approach would be to mobilise endogenous MSCs into the blood, thereby reducing costs and obviating regulatory and technical hurdles associated with development of cell therapies. However, pharmacological tools for MSC mobilisation are currently lacking. Here we show that β3 adrenergic agonists (β3AR) in combination with a CXCR4 antagonist, AMD3100/Plerixafor, can mobilise MSCs into the blood in mice and rats. Mechanistically we show that reversal of the CXCL12 gradient across the bone marrow endothelium and local generation of endocannabinoids may both play a role in this process. Using a spine fusion model we provide evidence that this pharmacological strategy for MSC mobilisation enhances bone formation.

## Introduction

A plethora of studies have reported that MSCs exhibit regenerative properties in a range of pre-clinical models of disease, including orthopaedic injuries.^1^ Consequently many clinical trials are now underway using culture-expanded MSCs as cell therapies injected intravenously or directly to the site of injury to promote tissue regeneration.^2^ In this context MSCs are thought to work primarily through release of paracrine regenerative factors, as MSCs do not appear to permanently integrate into damaged tissues.

A limited number of studies have explored whether MSCs, can—similarly to haematopoietic stem and progenitor cells (HSPCs)—be mobilised from the bone marrow into the blood. Thus, an increase in circulating MSCs has been reported in animal models of injury, and high-mobility group protein B1 (HMGB1) and substance P have been identified as the factors that mediate this response.^3–6^ Furthermore, an increase in circulating stromal cells has been reported in blood from patients following myocardial infarction^7,8^ or burns.^9^ In theory, increasing circulating numbers of MSCs would increase numbers that could be recruited to sites of tissue injury and thus accelerate or enhance tissue regeneration, as has been shown in the context of skin wound healing^3,10^ and bone fractures.^11^ Thus identifying drugs that efficiently mobilise these cells represents a potential regenerative therapy, obviating the need to harvest, expand and administer MSCs as required for the more traditional cell therapy approaches.

CXCR4 antagonists have been used to disrupt the CXCL12-CXCR4 retention axis to mobilise HSPCs from the bone marrow.^12–14^ We and others have shown that the CXCR4 antagonist, AMD3100 (Mozobil, Plerixafor), acts synergistically with G-CSF to mobilise HSPCs into the blood.^12,14,15^ This combination treatment is currently approved by the FDA for collection of HSPCs in patients with non-Hodgkin lymphoma and multiple myeloma and has provided a strategy to mobilise HSPCs in patients that are identified in the clinic as ‘poor mobilisers’.^13,16,17^ We have previously shown that G-CSF and/or a CXCR4 antagonist were not effective in terms of MSC mobilisation.^15^ However, we discovered that MSCs, but not HPCs, could be mobilised into the blood when a CXCR4 antagonist was administered to mice that had been pre-treated with vascular endothelial growth factor A (VEGF-A).^15^ Recently it has been reported that using this pharmacological strategy to mobilise MSCs enhances the rate of fracture healing in a rat model.^11^ This suggests that distinct mechanisms regulate the mobilisation of these discrete populations of progenitor cells and opens up the possibility of developing pharmacological tools to mobilise MSCs for clinical applications in the context of orthopaedic injuries.^18^

In this study we sought to identify pharmacological strategies to mobilise MSCs using clinically approved classes of drugs, as this would dramatically reduce the timeline and cost of translation. We show here that a β3 adrenergic receptor (β3AR) agonist, BRL37344, in combination with a CXCR4 antagonist (AMD3100/ Plerixafor) mobilises MSCs into the blood. Moreover, we show enhanced bone healing in an established rodent spine fusion model using this drug combination. Thus, we present a new combination treatment utilising two clinically approved classes of drugs for MSC mobilisation, which may have therapeutic applications in the context of orthopaedic injuries.

## Results

### Activation of βARs induces mobilisation of MSCs by a CXCR4 antagonist in mice

**Fig. 1:**
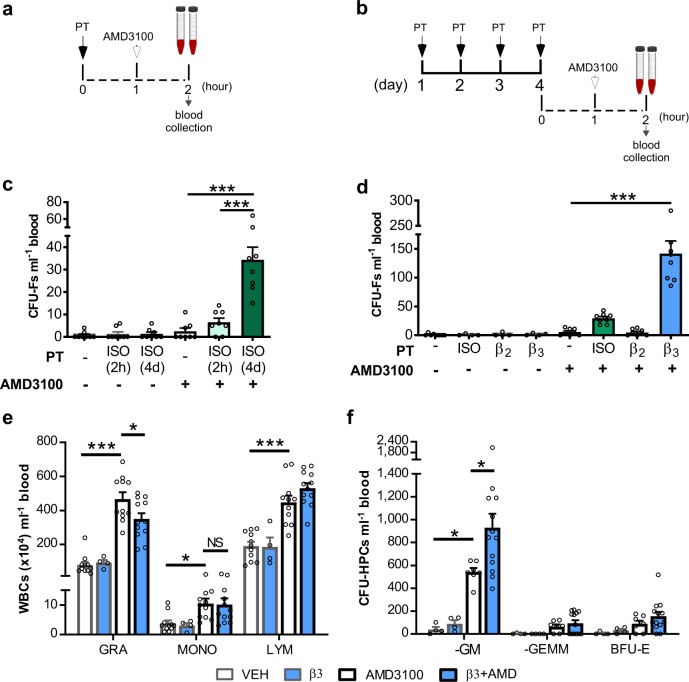
β3AR activation induces MSC mobilisation. **a**, **c** Experimental design for 2 h (2 h) of pretreatment (PT). Mice were pretreated with isoproterenol (ISO) or vehicle (−) followed 1 h later by the administration of AMD3100 or vehicle. **b**, **c** Experimental design for 4 days (4d) of PT. Mice were pretreated once daily for 4 days with ISO or vehicle. 1 h after the last injection, mice were administered AMD3100 or vehicle. In both treatment regimens, 1 h after AMD3100 or vehicle injection blood was collected for analysis of **c** circulating CFU-Fs; *n* = 8 mice per group. **d** Mice were pretreated (PT) with isoproterenol (ISO), clenbuterol (β2), BRL37344 (β3) or vehicle (−) once daily for 4 days. 1 h after the last injection, mice were administered AMD3100 or vehicle and 1 h later blood was collected for analysis of circulating **d** CFU-Fs. *n* = 4–8 mice per group. For BRL37344/AMD3100 treatment circulating **e** leucocytes (WBCs) and **f** CFU-HPCs were also analysed. *n* *=* 4–12 mice per group. **c**, **d** CFU-Fs and **f** CFU-HPCs are shown as colonies per ml of blood. Data of 2 independent experiments represented as mean ± s.e.m.; *P < 0.05, ***P < 0.001, NS not significant (one-way ANOVA with Bonferroni correction).

Initial experiments showed that treatment of mice with the CXCR4 antagonist, AMD3100, alone (as a single dose 1 h prior to blood collection) or the general βAR agonist (isoproterenol) administered either as a single dose (given 2 h prior to blood collection) (Fig. 1a) or as a daily dose for 4 days (Fig. 1b) did not stimulate the mobilisation of MSCs (as measured by CFU-Fs; Fig. 1c, d) into the blood. However, AMD3100 treatment following 4 days of isoproterenol significantly increased circulating numbers of CFU-Fs (Fig. 1c). Using the β2AR- and β3AR-specific agonists clenbuterol and BRL37344, respectively, alone or in combination with AMD3100 we show that CFU-F mobilisation is maximal after treatment with BRL37344 over 4 days followed by acute AMD3100 treatment (Fig. 1d). As seen for isoproterenol, the effect of the BRL37344 was only observed after the mice were pre-treated with the agonist for 4 days (Supplementary Fig. 1a). In addition to MSCs, AMD3100 treatment alone stimulated the robust mobilisation of granulocytes and lymphocytes and extremely low numbers of monocytes, as previously reported,^14^ importantly numbers of leucocytes were not further increased by pre-treatment with BRL37344 (Fig. 1e). In contrast, mobilisation of HPCs by AMD3100 was significantly enhanced by pre-treatment with BRL37344 (Fig. 1f).

### CFU-Fs mobilised into the blood exhibit characteristics of MSCs

**Fig. 2:**
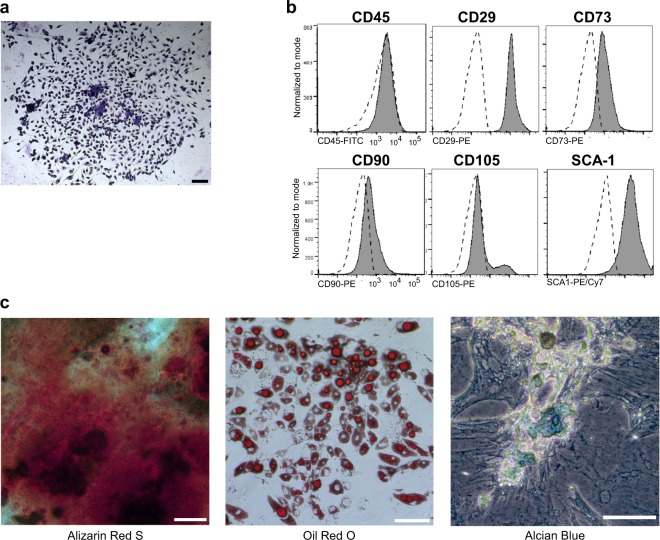
Characterisation of mobilised CFU-Fs. Mice were pretreated with BRL37344 (β3) once daily for 4 days. One hour after the last injection, mice were administered AMD3100 and 1 h later blood was collected for analysis of circulating (**a**–**c**) CFU-Fs. **a** Photomicrograph image depicts a blood mobilised CFU-F colony stained with Giemsa. **b** Histograms of surface marker expression on culture-expanded CFU-F cells as determined by flow cytometry. Shaded histograms represent marker expression and open dashed-line histograms represent fluorescence minus one (FMO) controls. **c** Panel shows trilineage differentiation staining of FACS sorted CD45^–^ culture-expanded CFU-Fs into osteocytes (Alizarin Red S), adipocytes (Oil Red) and chondrocytes (Alcian Blue). Scale bars, 100 µm. Experiment representative of *n* *=* 10 mice.

CFU-Fs mobilised into the blood following the BRL37344/AMD3100 treatment adhered to plastic and formed colonies (Fig. 2a). Following cell culture expansion of isolated CFU-Fs to obtain sufficient numbers for downstream assays, flow cytometry revealed that that these clonogenic cells expressed the classic surface markers of MSCs (CD29, CD90, CD73, CD105 and Sca-1) and were negative for CD45 (Fig. 2b). Furthermore, these cells could be differentiated in vitro into osteogenic, adipogenic and chondrogenic lineages using standard differentiation protocols (Fig. 2c). Both culture-expanded and endogenous MSCs are heterogeneous with no specific cell surface markers, making direct identification of these cells in the blood challenging. Murine MSCs with high clongenicity have been found to be enriched in PDGFRα^+^, Sca-1^+^, CD45^−^, Ter119^−^ cells (PαS cells).^19^ Direct analysis of blood revealed a trend towards increased numbers of PαS cells in the blood following BRL37344/AMD3100 treatment (Supplementary Fig. 2a). Further examination of receptor expression on circulating PαS cells and monocytes following BRL37344/AMD3100 treatment indicates that these PαS are distinct from monocytes, which express high levels of CD115 as compared with PαS cells (Supplementary Fig. 2b, c). Taken together these results indicate that the BRL37344/AMD3100 treatment mobilises a population of cells with fundamental characteristics of MSCs into the blood.

### β3AR agonists stimulates production of endocannabinoids and *N*-acyl ethanolamines in the bone marrow

**Fig. 3:**
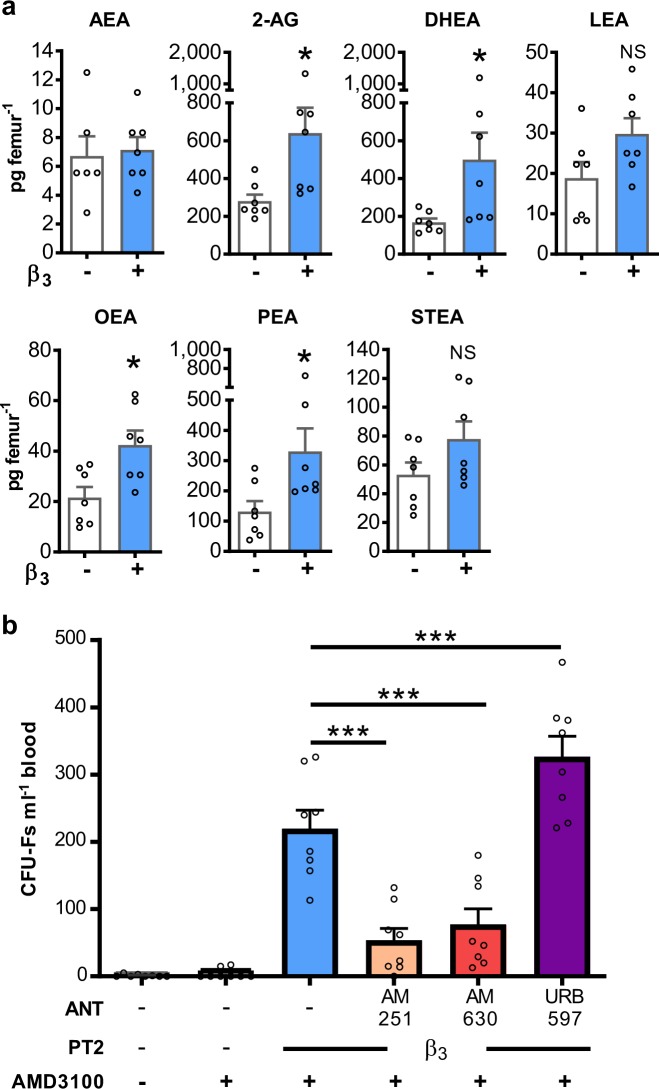
β3AR agonist treatment generates endocannabinoids and *N-acyl ethanolamines* in the bone marrow required for MSC mobilisation. **a** Mice were pretreated with URB597 in the presence or absence of BRL37344 (β3) once daily for 4 days. 2 h after the last injection, bone marrow was collected for endocannabinoid and *N*-acyl ethanolamine quantification by UPLC-MS/MS; *n* = 18–21 mice per group with bone marrow of 3 mice pooled together for analysis. **b** Mice were pretreated (PT) with BRL37344 (β3) or vehicle in the presence or absence of AM251 and AM630, CB1 and CB2 antagonists (ANT), respectively, or URB597 a FAAH inhibitor as indicated once daily for 4 days. One hour after the last injection, mice were administered AMD3100 or vehicle and 1 h later blood was collected for analysis of circulating CFU-Fs; *n* = 8 mice per group. CFU-Fs are shown as colonies per ml of blood. **a**–**b** Data of at least two independent experiments represented as mean ± s.e.m; *P < 0.05, ***P < 0.001*;* NS not significant. (**a** Student’s *t*-test and **b** one-way ANOVA with Bonferroni correction).

β3AR is expressed at high levels on adipocytes and β3AR-specific agonists are known to stimulate lipolysis in peripheral fat stores.^20,21^ In the bone marrow, adipocytes also express β3AR and contain high levels of triglycerides.^22–24^ Previous work has shown that infusion of isoproterenol into the femoral artery results in an increase in fatty acid amides (FFA) in the femoral vein,^25^ suggesting that β3AR activation stimulates lipolysis locally in the bone marrow. Recent studies have shown that β3AR agonists stimulate endocannabinoid production in white and brown adipose tissue in vivo.^26^ A mass spectrometry-based assay was employed, therefore, to directly examine whether endocannabinoids and/or *N*-acyl ethanolamines are generated in the bone marrow following 4 days of BRL37344 treatment. In these experiments, fatty acid amide hydrolase (FAAH) was inhibited by treatment of all mice with a specific inhibitor (URB597) to reduce the rapid hydrolysis of these lipid mediators. In mice in which only FAAH was inhibited, the endocannabinoids anandamide (AEA) and 2-arachidonoyl glycerol (2-AG), as well as several *N*-acyl ethanolamines (oleoyl ethanolamine (OEA), palmitoyl ethanolamine (PEA), docosahexaenoyl ethanolamine (DHEA), linoleoyl ethanolamine (LEA) and stearoyl ethanolamine (STEA)), could be detected in the bone marrow, with high basal levels of 2-AG, DHEA and PEA (Fig. 3a). Moreover, bone marrow levels of 2-AG, DHEA, OEA, and PEA were significantly increased following BRL37344 treatment (Fig. 3a). Our results demonstrate that β3AR-specific agonists can stimulate the generation of endocannabinoids and their congeners locally in the bone marrow.

### Activation of CB1 and CB2 is required for mobilisation of MSCs regulated by β3AR agonists

We next investigated whether these lipid-signalling molecules regulate the mobilisation of MSCs. Our data show that antagonists of both cannabinoid receptor 1 (CB1; AM251) or cannabinoid receptor 2 (CB2; AM630) significantly suppressed the BRL37344/AMD3100 mobilisation of CFU-Fs (Fig. 3b). This indicates that endocannabinoid signalling via CB1 and CB2 plays a role in this response.

The effects of lipid mediators are generally limited both spatially and temporally by enzymes that efficiently degrade and deactivate them. In the case of endocannabinoids, fatty acid amide hydrolase (FAAH) is key in their hydrolysis and inactivation.^27^ Therefore, we examined whether inhibition of FAAH with a specific inhibitor, URB597, would affect MSC mobilisation by BRL37344/AMD3100. Our results show that mobilisation of MSCs in response to BRL37344/AMD3100 was significantly enhanced following FAAH inhibition (Fig. 3b) consistent with endocannabinoids playing a role in this response.

To investigate whether the bone marrow was a potential source of mobilised MSCs we used the previously published in situ perfusion system of the femoral bone marrow^15^ (Supplementary Fig. 3a) and showed that infusion of AMD3100, directly into the vasculature of the bone marrow via cannulation of the femoral artery stimulates mobilisation of MSCs into the femoral artery in mice pre-treated with the BRL37344 and the FAAH inhibitor (Supplementary Fig. 3b).

### Bone marrow/blood CXCL12 chemokine gradient generated by AMD3100 mediates MSC mobilisation

**Fig. 4:**
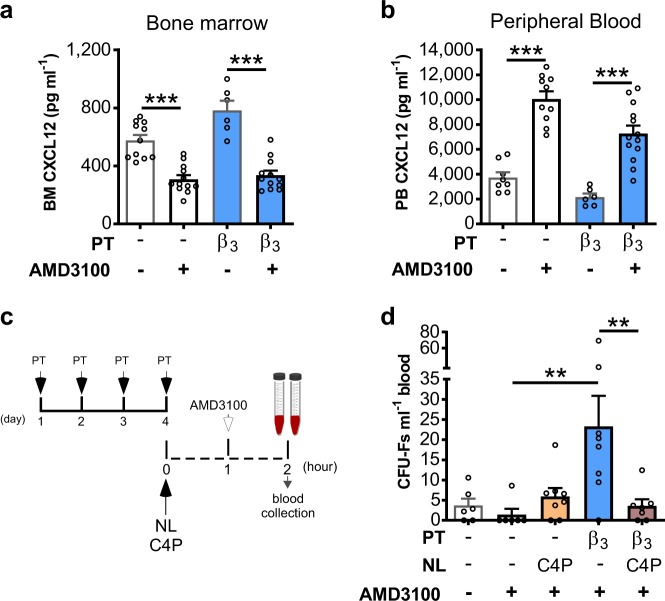
Neutralisation of CXCL12 chemokine gradient abrogates the mobilisation of MSCs in response to β3AR activation. **a**, **b** Mice were pretreated with BRL37344 (β3) or vehicle once daily on 4 consecutive days. One hour after the last injection, mice were administered AMD3100 and 1 h later femoral bone marrow and blood was collected for quantification of CXCL12 in **a** bone marrow (BM) supernatant and **b** peripheral blood (PB) plasma, respectively; *n* = 6–13 mice per group. CXCL12 levels are shown as pg per ml. **c**, **d** Experimental design; mice were pretreated (PT) with BRL37344 (β3) or vehicle once daily for 4 days. 1 h after the last injection, mice were administered AMD3100 in the presence or absence of chalcone 4-phosphate (C4P), a CXCL12 neutraligand (NL), and 1 h later blood was collected for analysis of **d** circulating CFU-Fs; *n* = 6–8 mice per group. CFU-Fs are shown as colonies per ml of blood. (**a**–**d**) Data of at least two independent experiments represented as mean ± s.e.m.; **P < 0.01,***P < 0.001 (one-way ANOVA with Bonferroni correction).

We have recently shown that in VEGF pre-treated mice AMD3100 mobilises MSCs into the blood by virtue of its ability to reverse the CXCL12 chemokine gradient across the bone marrow endothelium.^28,29^ We therefore investigated whether the same mechanism of action was operative in BRL37344 pre-treated mice. We show here that acute treatment with AMD3100 reversed the chemokine gradient across the sinusoidal endothelium, reducing levels of CXCL12 in the bone marrow (Fig. 4a) and increasing levels in the blood (Fig. 4b), to the same extent in mice treated with BRL37344 and the vehicle controls (Fig. 4a, b). A CXCL12 neutraligand, chalcone 4-phosphate (C4P)^30^ was used to investigate whether reversing the CXCL12 gradient was required for MSC mobilisation by BRL37344/AMD3100. Indeed treatment with chalcone 4-phospate abrogated MSC mobilisation stimulated by BRL37344/AMD3100 (Fig. 3c, d), suggesting that the ability of AMD3100 to reverse the gradient of CXCL12 across the sinusoidal endothelium is critical for MSC mobilisation.

### BRL37344 in combination with AMD3100 induces mobilisation of MSCs in rats

**Fig. 5:**
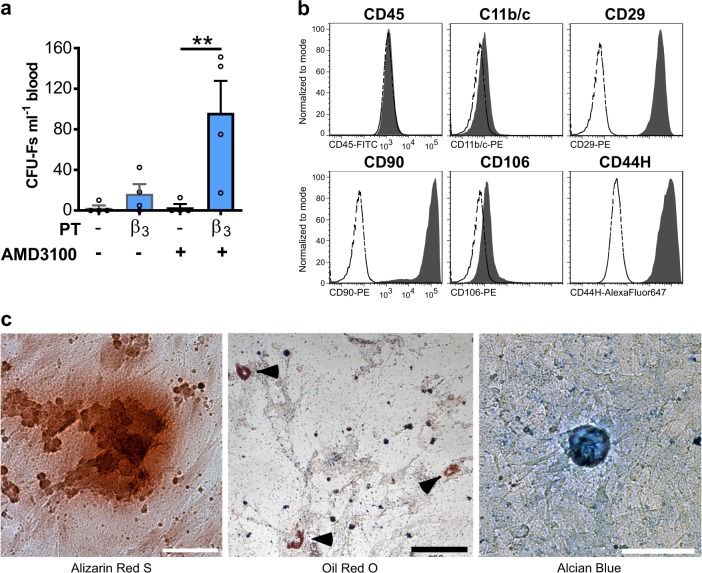
Characterisation of mobilised CFU-Fs in rats. **a**–**c** Rats were pretreated (PT) with BRL37344 (β3) or vehicle once daily for 4 days. One hour after the last injection, rats were administered AMD3100 or vehicle and 1 h later blood was collected for analysis of **a** circulating CFU-Fs; *n* = 4 rats per group. CFU-Fs are shown as colonies per ml blood. **b** Histograms of surface marker expression on culture-expanded rat CFU-F cells as determined by flow cytometry. Shaded histograms represent marker expression and open dashed-line histograms represent fluorescence minus one (FMO) controls. **c** Panel shows trilineage differentiation staining of culture-expanded, blood mobilised, rat CFU-Fs into osteocytes (Alizarin Red S), adipocytes (Oil Red) and chondrocytes (Alcian Blue). **a**–**c** Data of two independent experiments (**a**) represented as mean ± s.e.m; **P < 0.01 (one-way ANOVA with Bonferroni correction). Scale bars, 100 µm.

In order to investigate whether pharmacological mobilisation of MSCs could enhance bone formation it was first necessary to establish whether BRL37344/AMD3100 treatment mobilised MSCs in a rat model. As shown in Fig. 5 BRL37344/AMD3100 treatment of Lewis rats caused a significant increase in numbers of circulating CFU-Fs (Fig. 5a), which when expanded in culture were negative for CD45 and CD11b, but positive for CD29, CD90, CD106 and CD44H (Fig. 5b) and exhibited tri-lineage differentiation in vitro (Fig. 5c).

### Post-operative administration of β3AR agonist and CXCR4 antagonist enhances in vivo bone formation rate and new bone volume in an established rat posterolateral lumbar fusion model

Finally we investigated whether BRL37344/AMD3100 enhanced the rate of bone formation in an established rat posterolateral lumbar fusion model. For this study because we had detected some enhanced mobilisation of HPCs, in addition to MSCs, with BRL37344/AMD3100 (Fig. 1f), we compared the effects of BRL37344/AMD3100 to G-CSF/AMD3100, the gold standard for HPC mobilisation.

**Fig. 6:**
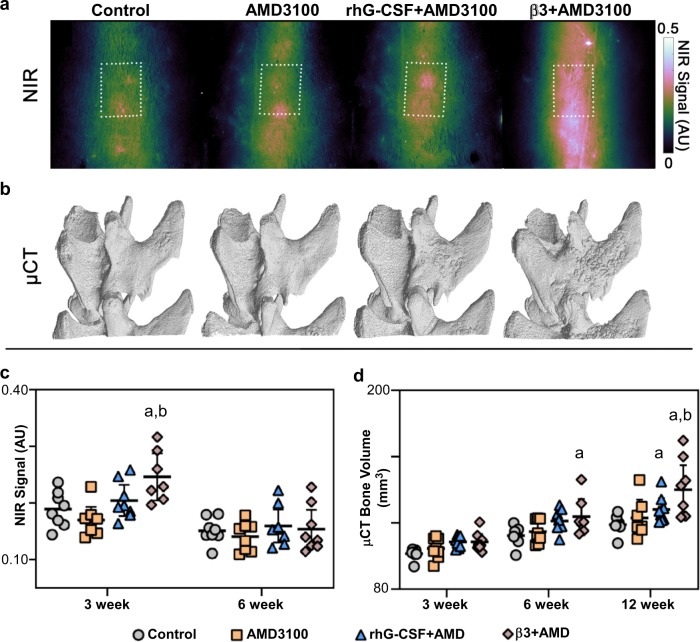
β3AR agonist and CXCR4 antagonist enhances in vivo bone formation. **a**–**d** Rats underwent bilateral posterolateral lumbar fusion surgery followed by rhG-CSF, BRL37344 or vehicle once daily for 4 days. Two hours after the last injection, rats were administered AMD3100 or vehicle. **a** Representative in vivo NIR fluorescence images of the L4/L5 fusion site at 3 weeks post op. **b** Representative 3D bone volumes at the L4/L5 fusion site, derived from in vivo µCT scans, at 12 weeks post op. Numerical results of **c** NIR fluorescence intensity, and **d** µCT-derived bone volume data. Data represented as individual data points with mean and 95% CI overlaid. *n* *=* 8 rats per group; **c** control vs β3 + AMD3100 ^a^*P* < 0.01, AMD3100 vs β3 + AMD3100 ^b^*P* < 0.001; **d** 6 weeks control vs β3 + AMD3100 ^a^*P* < 0.05, 12 weeks control/AMD vs β3 + AMD ^a,b^*P* ≤ 0.001. Twelve weeks control vs rhG-CSF + AMD3100 ^a^*P* < 0.05 (multiple comparison with Sidak post hoc correction).

The in vivo bone formation rate and volume of new bone formed were characterised in an established rat model of posterolateral lumbar fusion (PLF) by near-infrared (NIR) fluorescence and µCT imaging modalities (Fig. 6a, b), respectively. The rat model utilised demineralised bone matrix (DBM) as the graft material, which is a commonly utilised cell-free tissue product containing collagen and growth factors endogenous to bone. Stem cell mobilisation regimens were initiated 24 h after PLF surgery. The in vivo bone formation rate at 3 and 6 weeks post op was assessed by quantifying NIR fluorescence signal intensity of an IV-administered calcium chelating agent conjugated to an NIR fluorescent probe. BRL37344/AMD3100 treatment significantly increased NIR signal intensity at the L4–L5 level compared with both vehicle controls (*p* = 0.004) and AMD3100 alone (*p* < 0.001) at the 3-week timepoint (Fig. 6c). Compared to the 3-week timepoint, NIR signal intensity was decreased among all groups at 6 weeks with a lack of statistically significant differences. Live µCT-based measurement of new bone volume at the L4–L5 level demonstrated that BRL37344/AMD3100 animals formed a significantly higher volume of bone compared with vehicle controls at 6 weeks, and to both vehicle controls and AMD3100 alone at the 12-week endpoint (Fig. 6d). rhG-CSF/AMD3100 significantly increased new bone volume compared with saline controls at the 12-week endpoint. Only BRL37344/AMD3100 animals exhibited increased new bone volume at the L4–L5 level compared with vehicle controls and AMD3100 alone when all timepoints were taken into account.

## Discussion

Only a handful of studies have examined possible therapeutic benefits arising from circulating endogenous MSCs despite soaring numbers of pre-clinical and clinical trials currently using their culture-expanded counterparts as a cellular therapy (many administered systemically). Our study aimed to build from previous work^15,28^ where we hypothesised that pharmacological strategies stimulating the mobilisation of MSCs is an attractive approach to tissue regeneration—in particular in the context of orthopaedic injuries—as it circumvents a number of drawbacks faced during cellular therapy (e.g. culture modification and bias; high costs; and time intensive technical procedures).

In this study, we show that two drugs, a selective β3AR agonist, BRL37344, and the FDA-approved CXCR4 antagonist, AMD3100/Plerixafor, when used in combination mobilise MSCs into the blood. It is worth noting that the classical β3AR-specific agonist BRL37344 used throughout this study is recognised as a ‘rodent-selective’ agonist. Instead, the FDA-approved mirabegron^31^—currently used to treat overactive bladder—could be considered for human studies. At a molecular level we have de-lineated a pathway whereby MSC mobilisation is a two-step process firstly involving β3AR stimulated generation of endocannabinoids and *N-*acyl ethanolamines locally in the bone marrow. Secondly, the CXCR4 antagonist, AMD3100, acts by generating a gradient of CXCL12 across the bone marrow sinusoidal endothelium to promote migration of CXCR4-expressing MSCs into the blood.

β3AR is expressed at high levels on adipocytes and β3AR-specific agonists are known to stimulate lipolysis in peripheral fat stores. In the bone marrow, adipocytes also express β3AR and contain high levels of triglycerides.^22–24^ Furthermore, infusion of the general βAR agonist isoproterenol directly into the vasculature of the canine femur was shown to lead to the release of FFAs into the femoral vein, suggesting that lipolysis could also be stimulated locally in the bone marrow.^25^ Recent studies have shown that β3AR agonists stimulate endocannabinoid production in white and brown adipose tissue in vivo.^26^

Bone marrow adipose tissue expresses all of the biochemical machinery that is necessary to synthesise and degrade *N*-acyl ethanolamines and it has previously been reported that extracts of bone marrow contain *N*-acyl ethanolamines at similar levels to those reported in the brain, where they are known to have physiological effects.^32^ In this study, lipids extracted from the bone marrow were analysed by mass spectrometry and shown to contain endocannabinoids and *N*-acyl ethanolamines, with high basal concentrations of 2-AG, DHEA and PEA. Levels of these metabolites were shown to be significantly increased following BRL37344 treatment while other *N*-acyl ethanolamines, including the endocannabinoid AEA, remained unchanged. Therefore, we have provided direct evidence that systemic β3AR-specific activation drives the local generation of a unique profile of endocannabinoids and *N*-acyl ethanolamines in the bone marrow. This suggests that bone marrow adipose tissue can generate these bioactive lipid mediators. However, we cannot exclude the possibility that these lipid mediators are generated by other cells in the bone marrow, such as MSCs or adipocyte progenitors.

The function of endocannabinoids in the CNS has been well characterised and these lipid mediators are known to use a range of mechanisms to reduce the physical and the psychological effects of stress.^33^ Similarly, there is growing evidence that endocannabinoids are generated in the periphery in response to stress and that they function to combat the consequences of and to enhance the recovery from stress, for example, in response to injury.^34,35^ Our data suggest that endocannabinoids that are locally generated in the bone marrow regulate MSC activity and it will be interesting in the future to consider whether effects on bone marrow stem cells are part of the stress response. In this context, it has previously been shown that chronic stress stimulates an increase in noradrenaline levels in the bone marrow, which acts via β3AR receptors to stimulate HSPC mobilisation.^36^ It would be of interest to investigate whether the localised generation of endocannabinoids and *N*-acyl ethanolamines are also involved in this response.

The mechanism whereby these lipid mediators regulate MSC mobilisation is currently unknown. 2-AG is an endocannabinoid that acts as a full agonist of CB1 and CB2.^37^ In the periphery, and specifically in the bone, CB1 is primarily expressed on presynaptic sympathetic neurons and functions to reduce noradrenaline release,^38,39^ whereas CB2 is expressed on cells of the immune system and on osteoblasts.^32^ Our studies suggest that both of these receptors are involved in regulating the mobilisation of MSCs. Treatment of whole bone marrow, in vitro, with a CB2 agonist or 2-AG has been shown to increase the number and size of CFU-Fs.^40^ Evidence suggests that this effect is mediated indirectly through the action of CB2 agonists on an accessory cell, most likely macrophages.^40^ Macrophages are an important component of the stem cell niche and have previously been shown to play a critical role in regulating HPC mobilisation.^41,42^ We speculate, therefore, that the activation of BM macrophages by endocannabinoids could play a role in regulating MSC mobilisation. We cannot rule out direct effects of endocannabinoids on MSCs. Indeed, MSCs undergoing osteogenic differentiation have been shown to upregulate CB1 receptors on their surface and activation of CB1 receptors has been reported to positively affect their survival.^43^ PEA and DHEA do not mediate their effects via CB1 and CB2. In the central nervous system (CNS), DHEA has been reported to stimulate synaptogenesis and to enhance synaptic connectivity in a CB1- and CB2-independent manner;^44^ however, receptors for DHEA still remain to be identified. The receptor targets of PEA include the nuclear peroxisome proliferator-activated receptor-α (PPARα) and the cannabinoid-like G-coupled receptor GPR55, which are thought to facilitate many of its anti-inflammatory effects.^45^ Given that we have observed a significant increase in levels of 2-AG, DHEA and PEA in response to β3AR activation, it is possible that several distinct receptors, cell types and potential mechanisms regulate MSC mobilisation, an area that warrants further work.

MSC therapies have been investigated as a possible adjuvant to promote bone formation in the clinical settings of fracture repair and spinal arthrodesis. The results presented in this study show that BRL37344/AMD3100 mobilises MSCs with tri-lineage differentiation capacity into the blood, in mice and rats. To determine whether this pharmacological approach to increase circulating MSCs may be useful to promote bone tissue regeneration we assessed the effect of different pharmacological regimens in an established rat model of posterolateral lumbar fusion. In this study we compared treatment with AMD3100 alone or in combination with G-CSF or BRL3744. G-CSF/AMD3100 was used as it is the gold standard for HSPC mobilisation in the clinic. However, only the BRL37344/AMD3100 treatment significantly increased early evidence of in vivo mineralisation as well as new bone volume formation compared with vehicle and AMD3100 alone. This in vivo data suggests that MSC mobilisation via β3AR agonism and CXCR4 antagonism could be a useful adjunct treatment to enhance the bone-forming potential of existing bone graft materials.

It is important to note, however, that while the rat posterolateral lumbar fusion model employed here is an established early phase model used to assess in vivo bone formation associated with pharmacologic and/or biomaterial strategies, a large animal study is a critical next step in validation of this pharmacological strategy to enhance bone formation and the potential of translation of these findings into man. Moreover, the rat posterolateral lumbar fusion experiment presented in this study only examined the efficacy of the mobilisation therapy as provided in the acute phase after surgery. Future work should examine the impact of repeat dosing, especially during the later anabolic and re-modeling phases of bone formation.

Taken together, our data provide proof of concept that pharmacological strategies that mobilise MSCs into the blood can enhance bone repair. AMD3100 is a FDA-approved drug, Plerixafor, while a human specific β3AR agonist, Mirabergon, is also FDA approved, suggesting that these findings could be rapidly translated to the clinic. Moreover, they pave the way to explore whether this drug combination can promote tissue regeneration in the context of other orthopaedic injuries.

## Methods

### Animals

Experiments were carried out on female BALB/c mice (ages 10–14 weeks, 22–25 g) which were purchased from Harlan Laboratories. Female Lewis rats weighing 200–250 g were purchased from Charles River Laboratories. All studies were carried out under the United Kingdom’s Animals (Scientific Procedures) Act of 1986 and local ethical approval from Imperial College London. The rat posterolateral lumbar fusion procedure was performed in accordance with an approved protocol reviewed and administered by Beaumont Health’s IACUC, which conformed with applicable federal and local regulations.

### Administration of drugs

Mice (or rats) were administered the β adrenergic agonists isoproterenol (10 mg/kg i.p.), clenbuterol (5 mg/kg i.p.) and BRL37344 (10 mg/kg i.p.), or vehicle either on 4 consecutive days or 2 h prior to the cull. One hour after the last injection, mice were administered the CXCR4 antagonist AMD3100 (5 mg/kg i.p.) or vehicle. One hour later their blood was collected via cardiac puncture for enumeration of circulating CFU-Fs. The antagonists or inhibitors AM251 (5 mg/kg i.p.), AM630 (5 mg/kg i.p.), URB597 (0.5 mg/kg i.p.), or vehicle, were administered 60 min prior to the injection of the mobilising treatment, BRL37344. The CXCL12 neutraligand Chalcone 4-phosphate (1.5 µmol/kg i.v.) was administered 60 min prior to the injection of AMD3100 on day 4. 24 h after posterolateral lumbar fusion surgery, rats were administered rhG-CSF (100 µg/kg s.c.), BRL37344 (10 mg/kg s.c.), or vehicle on 4 consecutive days. Two hours after the last injection, rats were administered AMD3100 (5 mg/kg s.c.).

A list of all the drugs used in this study and the companies at which they were purchased, including information regarding the vehicle, is found in Supplementary Table 1.

### CFU-F assay

Harvested peripheral blood (or perfusate; Supplementary Fig. 3) was red blood cell-lysed and 1 × 10^6^ cells were added to tissue culture-treated six-well plates containing 3 ml of Mesencult^TM^ (05502; StemCell Technologies) to select for CFU-Fs. Cultures were incubated at 37 °C and media was changed on day 7. Blood- and perfusate-derived CFU-Fs were enumerated on day 21. Blood derived-CFU-Fs were further expanded and assessed for mesenchymal lineage markers by flow cytometry and following CD45 depletion (flow cytometry sorting of CD45 negative cells, exclusively for mouse cultures) cells were prepared for mesenchymal trilineage differentiation.

### Leucocyte and CFU-HPC enumeration

Harvested peripheral blood (before RBC lysis), ~4 μl, was placed on one end of the glass slide, and was immediately spread along its length. The thin film of blood was fixed with methanol, and subsequently stained with hematoxylin and eosin (H&E); ready for leucocyte (WBC) differential counts under the light microscope. Harvested peripheral blood was red blood cell-lysed and 1 × 10^5^ cells were added to tissue culture-treated petri dishes containing 1 ml of MethocultTM (M3434; StemCell Technologies) to select for CFU-HPCs. Cultures were incubated at 37 °C and quantified on day 12.

### Flow cytometry analysis and fluorescence-activated cell sorting

Flow cytometry was used to determine the expression of MSC markers on blood CFU-Fs that had been expanded in culture for a maximum of three to four passages. Cells were stained with fluorochrome-conjugated monoclonal antibodies directed against CD45, CD11b/c, CD29, CD73, CD90, CD105, CD106, CD44H and Sca-1. Cells were analysed using a Fortessa flow cytometer (Becton Dickson). For the murine cells fluorescence-activated cell sorting was performed on blood CFU-Fs expanded in culture, before performing mesenchymal trilineage differentiation. Thus cells were stained with fluorochrome-conjugated monoclonal antibodies directed against CD45. CD45 negative cells were sorted using a FACS Aria II (Becton Dickson) and then grown to confluence to perform differentiation assays.

Harvested peripheral blood (pooled *n* = 4–5 mice) was red blood cell-lysed and circulating PαS cells were enumerated and characterised using flow cytometry. Cells were stained with fluorochrome-conjugated monoclonal antibodies directed against CD45, CD119, CD31, PDGFRα, SCA-1, CXCR4, and CD115. A list of all the antibodies used in this study and the companies at which they were purchased is in Supplementary Table 2.

### Mesenchymal trilineage differentiation

Blood mobilised CFU-F colonies were first expanded, before directed differentiation was initiated. These cells were then cultured for 3 or 4 weeks in adipogenic, osteogenic and chondrogenic differentiation media. Adipogenic medium contained 0.5 µM dexamethasone, 0.5 µM isobutyl-methylxanthine and 50 µM indomethacin. Osteogenic medium contained 1 nM dexamethasone, 20 µM β-glycerolphosphate and 50 mM ascorbic acid-2-phosphate. Serum-free chondrogenic medium contained 10 ng/ml TGF-β1, 100 nM dexamethasone, 50 µg/ml ascorbic acid-2-phosphate, 100 µg/ml sodium pyruvate, 40 µg/ml l-proline, 1X ITS + 3 (Sigma-Aldrich) and 1.25 mg/ml BSA. Cells were then stained with Oil Red O, Alizarin Red S and Alcian Blue for adipocytes, osteoblasts and chondrocytes, respectively.

Photomicrograph imaging was performed on Zeiss AxioVert 200 inverted microscopes (Carl Zeiss) mounted with Hamamatsu cameras (ORCA-ER and EM-CCD; Hamamatsu Photonics K.K.).

### UPLC-MS/MS analysis of endocannabinoids and *N*-acyl ethanolamines

Bone marrow flush (pool of three mice) was processed in order for the extraction of endocannabinoids and *N*-acyl ethanolamines.^46,47^ Samples were analysed on a triple quadrupole mass spectrometer (Xevo TQ-S, Waters) with electrospray ionisation probe coupled to an ultra-high performance liquid chromatography pump (UPLC, Acquity, Waters) (UPLC-MS/MS; the analytes were quantified via multiple reaction monitoring assays using deuterated internal standards and internal calibration lines. Details concerning extraction and analysis can be found in the Supplementary Methods.

### CXCL12 enzyme-linked immunosorbent assay

CXCL12 content in blood plasma and bone marrow supernatant was quantified using CXCL12 capture (MAB350) and detection (BAF351) antibodies (RnD systems), and the assay was performed according to the manufacturers’ instructions. Recombinant mouse CXCL12 (RnD systems) was used to generate a standard curve.

### Rat posterolateral lumbar fusion surgical procedure

Under IACUC approval, female Lewis rats (14 weeks, ~200 g) underwent bilateral posterolateral lumbar fusion of the L4/L5 motion segment, as previously described. Briefly, anaesthetised rats were placed prone on a heated surgical bed and the hair from the lumbar region was clipped and the skin sterilely prepared. A midline skin incision was made over the lumbar spine and two separate fascial incisions were made to gain access to the L4–L5 level, which was confirmed with intra-operative fluoroscopy. After blunt dissection, the L4 and L5 transverse processes were bilaterally decorticated using a microdrill equipped with a 0.4 mm burr. Demineralised bone matrix (AlloFuse DBM Putty, AlloSource Inc., Centennial, CO) was utilised as the graft material and was placed bilaterally in a bridging fashion across the L4–L5 intertransverse process space (0.5 cc of DBM per side). The surgical site was closed in layers and animals were recovered from anaesthesia. Following surgery, rats were randomised to the priming and mobilisation regimens described above, with the first injection given 24 h post op (eight rats per group). Rats were allowed ad libitum cage activity before and after surgery, fed a standard diet, and group housed in a 12 h light/dark facility. At 12 weeks post op, rats were euthanized and lumbar spines were aseptically harvested.

### In vivo near-infrared fluorescence imaging of active mineralisation

At 3- and 6-weeks post op, a 10 nmol dose of IRDye 680RD BoneTag (LI-COR Biosciences, Lincoln, NE) was intravenously administered to rats via tail vein injection. BoneTag is a calcium chelating agent conjugated to a near-infrared (NIR) fluorescent probe, which is actively incorporated into newly forming bone, enabling quantitative biochemical imaging of bone formation rate. Rats were anaesthetised via inhaled isoflurane and underwent NIR fluorescence imaging immediately before and 24-h after BoneTag injection at each time point. Planar fluorescence images were acquired in the 700 nm channel (LI-COR Pearl Impulse, Lincoln, NE). A rectangular region-of-interest around L4/L5 was generated, and the NIR fluorescence signal intensity within this region-of-interest was quantified. Intensity values were normalised to adjacent skin.

### Longitudinal micro-computed tomography of in vivo bone formation

At 3-, 6- and 12-weeks post op, rats underwent in vivo micro-computed tomography (µCT, VivaCT-80, Scanco Medical, Brüttisellen, Switzerland) imaging to quantify bone volume at the fusion site. Animals were maintained under isoflurane anaesthesia during imaging, which was performed at 55 kVp, 145 µA, 450 ms integration time and isotropic voxel size of 31.2 µm. Each µCT scan was re-oriented to ensure consistent alignment. Bone volume at the fusion site was computed within a manually-determined volume-of-interest, encompassing the posterior elements and facets of the L4 and L5 vertebral bodies and bounded caudally by the bottom of the L4–L5 caudal endplate, which was generated via a custom MATLAB algorithm.

### Statistical analysis

Analyses of data were performed using GraphPad Prism 5 and 8. Data are represented as the mean and standard error of the mean (mean ± s.e.m.) displaying individual data points, and statistical analysis was performed using unpaired Student’s *t*-test or one-way analysis of variance (ANOVA) with Bonferroni’s post-test. Results were considered statistically significant when *P* < 0.05 and represented using asterisks (*P < 0.05; **P < 0.01; ***P < 0.001). The number of mice (or rats) used and independent experiments performed are stated in the figure legends. *n* = number of mice (or rats) per group. Between-group differences in in vivo NIR fluorescence intensity and µCT-quantified bone volume were analysed via a mixed effects model with one between-subject effect (treatment group) and one within-subject effect (time). A compound symmetry covariance structure was used, based on preliminary optimisation. For all comparisons, post hoc Sidak tests were performed to adjust for multiple comparisons, and *P* < 0.05 was considered statistically significant.

### Reporting summary

Further information on research design is available in the Nature Research Reporting Summary linked to this article.

## Supplementary information

Supplementary material

Reporting Summary

## Data Availability

All data sets associated with the current study are available from the corresponding author on reasonable request.
