# Supplementary material for: Pharmacological tools to mobilise mesenchymal stromal cells into the blood promote bone formation after surgery
Source: NPJ Regen Med. 2020 Feb 21;5:3. doi: 10.1038/s41536-020-0088-1 (PMC7035363; doi:10.1038/s41536-020-0088-1)
Supplement: Supplementary file 1 — Supplementary material [file 41536_2020_88_MOESM1_ESM.pdf]

Supplementary Information

Pharmacological tools to mobilize mesenchymal stromal cells into the blood promote bone formation after surgery

Authors: Tariq G. Fellous*, Andia N. Redpath*, Mackenzie M. Fleischer, Sapan Gandhi, Samantha E. Hartner, Michael D. Newton, Moïra François, Suet-Ping Wong, Kate H. C. Gowers, Adam M. Fahs, Daniel R. Possley, Dominique Bonnet, Paula Urquhart, Anna Nicolaou, Kevin C. Baker, and Sara M. Rankin.

*co-first authors

Supplementary Figures

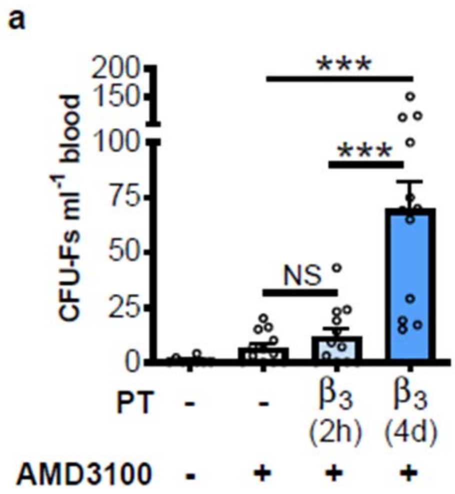

Supplementary Figure 1: β3AR activation mediates the mobilization of MSCs; cell kinetics.
(**a**) Mice were pretreated (PT) either 2 hours (2h) or once daily for 4 days (4d) with BRL37344 (β3) or vehicle (-). 1 hour after the last injection, mice were administered AMD3100 or vehicle, and 1 hour later blood was collected for analysis of circulating CFU-Fs; *n*= 8-12 mice per group. CFU-Fs are shown as colonies per ml blood. Data of at least 2 independent represented as mean ± s.e.m; ****P*< 0.001*;* NS, not significant (one-way ANOVA with Bonferroni's correction).

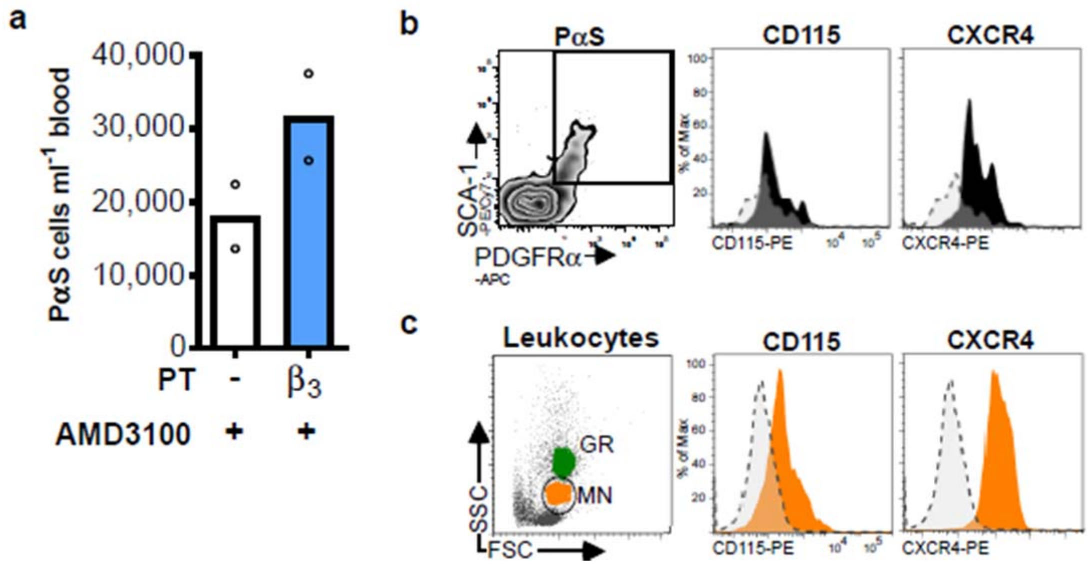

Supplementary Figure 2: β3AR activation mediates the mobilization of PαS cells.
(**a-c**) Mice were pretreated with BRL37344 (β3) or vehicle once daily for 4 days. 1 hour after the last injection, mice were administered AMD3100 and 1 hour later blood was collected for analysis of circulating (**a-b**) PαS cells ($CD45^-CD119^-CD31^-PDGFRa^+SCA\text{-}1^+$) and (**c**) leukocytes ($CD119^-CD45^+$). *n*= total of 8 mice/group; 4 mice pooled/sample. Histograms of surface marker expression on blood (**b**) PαS cells and (**c**) monocytes as determined by flow cytometry. Shaded histograms represent marker expression and open dashed-line histograms represent fluorescence minus one (FMO) controls. Data of 2 independent experiments represented as mean.

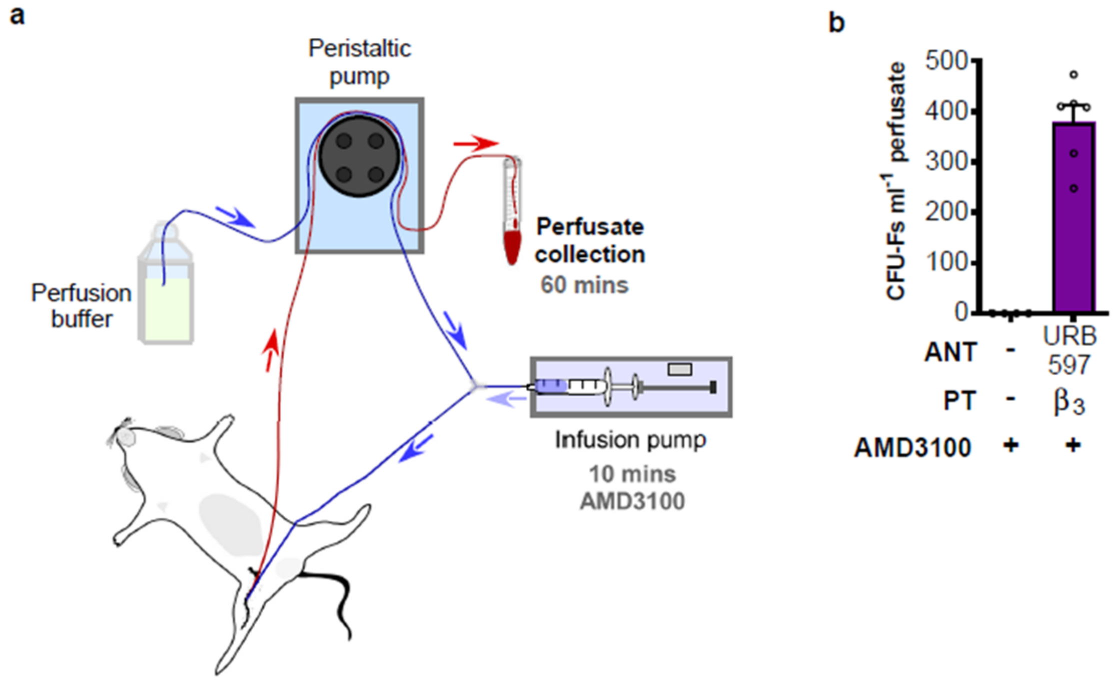

Supplementary Figure 3: Direct mobilization of MSCs from the femoral bone marrow.
Mice were pretreated with URB597, BRL37344 or vehicle (-) once daily for 4 days as described in the supplementary methods section. 1 hour after the last injection, mice underwent perfusion of the right hind limb. (**a**) The hind limb was infused with AMD3100 or vehicle for 10 minutes and further perfused for 50 minutes while collecting the perfusate for analysis of (**b**) CFU-Fs. CFU-Fs are shown as colonies per ml perfusate. *n*= 4-6 mice per group. Data of 2 independent experiments represented as mean ± s.e.m.

Supplementary Methods

Materials

The source, dose and vehicle for all drugs is shown in supplementary table 1. Mesencult proliferation kit (StemCell Technologies). SDF-1α/CXCL12 ELISA (R&D Systems). Commercially available standards for anandamide (AEA), AEA-*d8*, palmitoylethanolamide (PEA), docosahexaenoyl ethanolamide (DHEA), α-linoleoyl ethanolamide (ALEA), oleoyl ethanolamide (OEA), stearoyl ethanolamide (STEA), linoleoyl ethanolamide (LEA), 2-arachidonyl glycerol (2-AG), 2-AG-*d8* were purchased from Cayman Chemical Co (Ann Arbour, MI, USA). Ultrapure water was tapped by a Purelab Flex system from Elga Process Water (Marlow, UK), Strata Si-1 (Silica) SPE cartidges (Phenomenex, Macclesfield, UK). Chloroform and methanol (Chromosolv LC-MS grade - Sigma-Aldrich). Acquity UPLC® BEH Phenyl C18 column (1.7µm, 2.1 x 50 mm) and Acquity UPLC® BEH Phenyl VanGuard pre-column (1.7 µm, 2.1 x 5 mm) were from Waters.

Supplementary Methods Table 1: *In vivo* reagents and solvents/vehicles.

**Reagent**	**Company**	**Dose (*In Vivo*)**	**Vehicle**
Isoproterenol	Sigma Aldrich	10 mg/kg	Sterile dH_20
Clenbuterol	Sigma Aldrich	5 mg/kg	Sterile dH_20
BRL37344	Sigma Aldrich	10 mg/kg	Sterile dH_20
AMD3100	Sigma Aldrich	5 mg/kg	PBS
AM251	Tocris Bioscience	5 mg/kg	PBS v/v 6.25% EthOH & 5% Tween 80
AM630	Tocris Bioscience	5 mg/kg	PBS v/v 6.25% DMF & 5% Tween 80
URB597	Sigma Aldrich	0.5 mg/kg	PBS v/v 2.5% EthOH
Chalcone 4-phosphate	Kindly donated by Dr Dominique Bonnet (Chargé de Recherche au CNRS, Laboratoire d'Innovation Thérapeutique, France).	1.5 µmol/kg	PBS
rhG-CSF	Filgrastim, Amgen Inc., Thousand Oaks, CA.	100 µg/kg	saline

Supplementary Methods Table 2: For flow cytometry antibodies.

Antibody	Clone	Company
**Anti-Mouse**		
CD29-PE	eBioHMb1-1	eBioscience
CD45-FITC	30-F11	BD
CD73-PE	TY/11.8	Biolegend
CD90.2-PE	30-H12	Biolegend
CD105-PE	M17/18	eBioscience
Ly-6A-E/Sca1-PECy7	D7	BD
CD45 - PB	30-F11	Invitrogen
CD45 – BV510	30-F11	Biolegend
CD31 - PB	390	Invitrogen
CD31 - FITC	390	Biolegend
PDGFRα - APC	APA5	eBioscience
TER119 – V450	TER-119	BD
CD115-PE	ASF98	eBioscience
CXCR4 - PE	L276F12	Biolegend
**Anti-rat**		
CD106-PE	MR106	BioLegend
CD90-PE	KW322	BioLegend
CD45-FITC	OX-1	BioLegend
CD44H-AlexaFluor 647	OX-49	BioLegend
CD11b/c-PE	OX-42	BioLegend
CD29-PE	HMβ1-1	BioLegend

Supplementary Methods Figure 1: Gating strategy examples.

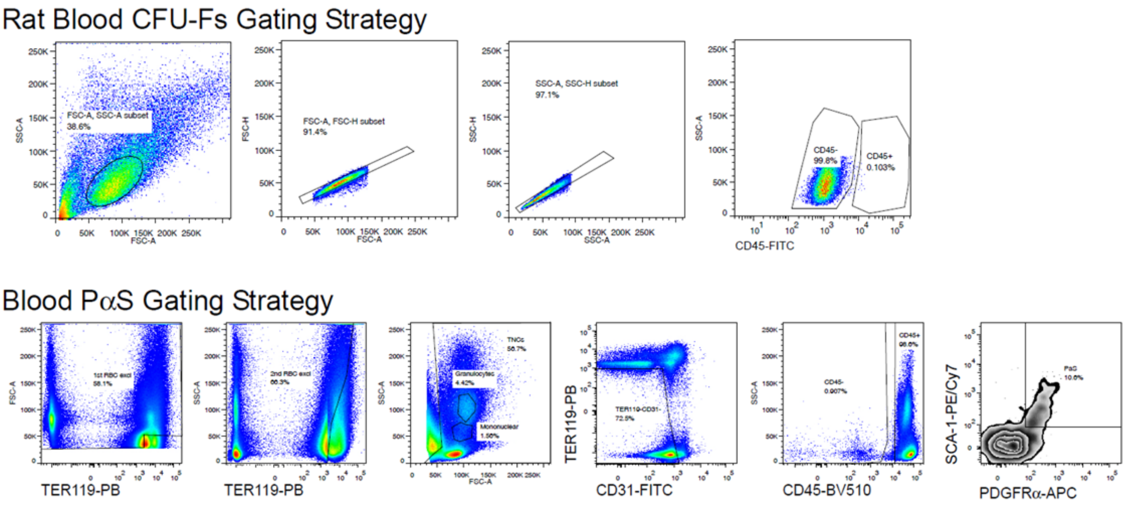

Extraction of endocannabinoids and *N*-acyl ethanolamines from mouse plasma and bone marrow flush

Bone marrow flushed from the femurs of 3 mice were pooled (6ml in total) per treatment. This volume was required to achieve sufficient concentrations of endocannabinoids and *N*-acyl ethanolamines for detection by UPLC/ESI-MS/MS. Extraction of lipids was carried out by addition of chloroform : methanol (2:1, v/v) [1, 2]. Specifically, ice cold chloroform : methanol was added to each bone marrow supernatant (ml per pooled bone marrow sample), followed by the internal standards AEA-*d*8 (20 ng) and 2AG-*d*8 (40 ng). The resulting suspensions were kept on ice for 30 min with occasional vortexing and centrifuged at 5000 rpm for 8 min, to separate the organic and aqueous phases. The organic layer (bottom) from each sample was then removed into a clean vial. The supernatant was evaporated under a fine stream of nitrogen. Once dried the extract was reconstituted in 1 ml of chloroform and semi-purified by solid phase exctraction (SPE) [3]. Briefly, the silica cartridge was equilibrated with 5 x 1 ml cholorform, the extract was applied, the cartridge was washed with 2 x 1 ml chloroform and the endocannabinoids and *N*-acyl ethanolamines eluted with 5 x 1 ml chloroform:methanol (2:1 v/v). The remaining residue, reconstituted in 100 µl ethanol was stored at -20°C, for no more than 1 week, awaiting UPLC-MS/MS analysis.

UPLC-MS/MS analysis

All analyses were performed on triple quadrupole mass spectrometer equipped with an electrospray ionization probe (Xevo TQ-S Waters) and coupled to an ultra-high performance chromatography pump (UPLC; Acquity, Waters). The system was controlled by MassLynx v4.1 Software. TargetLynx was used to calculate the concentration of analytes of interest using deuterated internal standards and internal calibration lines. All analytes were monitored on the positive ionisation mode. Capillary voltage was set at 2000V, source temperature at 150°C, desolvation temperature at 400°C and the cone voltage at 20 V. Multiple reaction monitoring (MRM) transitions as published before [1, 2].

Chromatographic analysis of the endocannabinoids and congeners was performed on an Acquity UPLC® BEH Phenyl C18 column (1.7µm, 2.1 x 50 mm; Waters) maintained at 25°C supported with Acquity UPLC® BEH Phenyl VanGuard pre-column (1.7 µm, 2.1 x 5 mm; Waters). Sample injections were performed with the Acquity sample manager (Waters); the sample chamber temperature was set at 8°C. The injection volume was 3 µL and the flow rate 0.6 ml/min. Analytes were separated using an acetonitrile-based gradient system comprising two solvents: solvent A: water/ glacial acetic acid 99.5:0.5 (v/v); solvent B: acetonitrile/glacial acetic acid 99.5:0.5 (v/v). The following gradient was used: Initial conditions 22% solvent B increasing linearly to 28% solvent B at 3min; 3.0-3.1 increase of solvent B to 55 % and remaining at 55% up to 10.9 min; 10.9-11.0 min increase of solvent B to 80% and remaining at 80% up to 12.5 min; 12.5-12.51 decrease of solvent B to 22%.

In situ perfusion of mouse hind limb

Mice were administered URB597 (0.5 mg/kg i.p.), BRL37344 (10 mg/kg i.p.) or vehicle on 4 consecutive days. 1 hour after the last injection, the mice were anaesthetized. The femoral vein and artery were exposed and cannulated in situ immediately after the hind limb was isolated by occlusion of the surrounding arteries as previously described [4, 5]. Perfusion buffer was infused via the arterial cannula and removed from the venous cannula using a Minipuls Peristaltic Pump (Anachem). The hind limb was initially infused with AMD3100 (0.1 mM) for 10 minutes using an infusion/withdrawal pump (Harvard Instruments) and was further perfused for 50 minutes with perfusion buffer [6]. The perfusate was collected over 60 minutes and then centrifuged and resuspended in DMEM (Gibco) + 20% fetal bovine serum for enumeration of cells.

Supplementary references

1. Felton, S.J. *et al.* Serum endocannabinoids and N-acyl ethanolamines and the influence of simulated solar UVR exposure in humans in vivo. *Photochem Photobiol Sci* **16**, 564-574 (2017).
2. Kendall, A.C. *et al.* Distribution of bioactive lipid mediators in human skin. *J Invest Dermatol* **135**, 1510-1520 (2015).
3. Guo, L., Amarnath, V. & Davies, S.S. A liquid chromatography-tandem mass spectrometry method for measurement of N-modified phosphatidylethanolamines. *Analytical biochemistry* **405**, 236-245 (2010).
4. Pitchford, S.C., Furze, R.C., Jones, C.P., Wengner, A.M. & Rankin, S.M. Differential mobilization of subsets of progenitor cells from the bone marrow. *Cell stem cell* **4**, 62-72 (2009).
5. Pitchford, S.C., Hahnel, M.J., Jones, C.P. & Rankin, S.M. Troubleshooting: Quantification of mobilization of progenitor cell subsets from bone marrow in vivo. *Journal of pharmacological and toxicological methods* **61**, 113-121 (2010).
