# Supplementary material for: Pharmacological tools to mobilise mesenchymal stromal cells into the blood promote bone formation after surgery
Source: NPJ Regen Med. 2020 Feb 21;5:3. doi: 10.1038/s41536-020-0088-1 (PMC7035363; doi:10.1038/s41536-020-0088-1)
Supplement: Supplementary file 2 — Reporting Summary [file 41536_2020_88_MOESM2_ESM.pdf]

PROF SARA RANKIN

nature research

Corresponding author(s): DBPR: Manuscript Number: NPJREGENMED-00328R

Last updated by author(s): 16/01/20

Reporting Summary

Nature Research wishes to improve the reproducibility of the work that we publish. This form provides structure for consistency and transparency in reporting. For further information on Nature Research policies, see Authors & Referees and the Editorial Policy Checklist.

Statistics

For all statistical analyses, confirm that the following items are present in the figure legend, table legend, main text, or Methods section.

n/a	Confirmed	
☐	☒	The exact sample size (n) for each experimental group/condition, given as a discrete number and unit of measurement
☐	☒	A statement on whether measurements were taken from distinct samples or whether the same sample was measured repeatedly
☐	☒	The statistical test(s) used AND whether they are one- or two-sided<br>*Only common tests should be described solely by name; describe more complex techniques in the Methods section.*
☐	☒	A description of all covariates tested
☐	☒	A description of any assumptions or corrections, such as tests of normality and adjustment for multiple comparisons
☐	☒	A full description of the statistical parameters including central tendency (e.g. means) or other basic estimates (e.g. regression coefficient) AND variation (e.g. standard deviation) or associated estimates of uncertainty (e.g. confidence intervals)
☒	☐	For null hypothesis testing, the test statistic (e.g. F, t, r) with confidence intervals, effect sizes, degrees of freedom and P value noted<br>*Give P values as exact values whenever suitable.*
☒	☐	For Bayesian analysis, information on the choice of priors and Markov chain Monte Carlo settings
☒	☐	For hierarchical and complex designs, identification of the appropriate level for tests and full reporting of outcomes
☒	☐	Estimates of effect sizes (e.g. Cohen's d, Pearson's r), indicating how they were calculated

Our web collection on statistics for biologists contains articles on many of the points above.

Software and code

Policy information about availability of computer code

Data collection	Zen 2.3 Blue, BD FACS Diva, MassLynx v4.1 (Waters), Magellan.
Data analysis	GraphPad Prism 5 and 8, MS Excel Professional Plus 2013, FlowJo v7 and v10, Matlab, Zen 2.3 Blue, BD FACS Diva, MassLynx v4.1 (Waters).

For manuscripts utilizing custom algorithms or software that are central to the research but not yet described in published literature, software must be made available to editors/reviewers. We strongly encourage code deposition in a community repository (e.g. GitHub). See the Nature Research guidelines for submitting code & software for further information.

Data

Policy information about availability of data

All manuscripts must include a data availability statement. This statement should provide the following information, where applicable:

- Accession codes, unique identifiers, or web links for publicly available datasets
- A list of figures that have associated raw data
- A description of any restrictions on data availability

All data sets associated with the current study are available from the corresponding author on reasonable request.

Field-specific reporting

Please select the one below that is the best fit for your research. If you are not sure, read the appropriate sections before making your selection.

☒ Life sciences ☐ Behavioural & social sciences ☐ Ecological, evolutionary & environmental sciences

Life sciences study design

All studies must disclose on these points even when the disclosure is negative.

Sample size	Describe how sample size was determined, detailing any statistical methods used to predetermine sample size OR if no sample-size calculation was performed, describe how sample sizes were chosen and provide a rationale for why these sample sizes are sufficient.
Data exclusions	Data exclusion was exercised to a minimum, and was only applied in cases of clear experimental issues (e.g. bacterial/yeast contamination of primary cells/colonies, values outside of standard curves or exceeded detection capacity of assay) or statistical outliers when detected (GraphPad).
Replication	Experiments were carried out at least two independent times with a good sample size in each group to ensure replication. Independent researchers were also involved in various experiments to ensure replication between different users.
Randomization	Mice and rats were randomly sorted into groups.
Blinding	Blinding was exercised when possible, such as data collection was carried out without knowledge of treatment condition for each sample.

Reporting for specific materials, systems and methods

We require information from authors about some types of materials, experimental systems and methods used in many studies. Here, indicate whether each material, system or method listed is relevant to your study. If you are not sure if a list item applies to your research, read the appropriate section before selecting a response.

Materials & experimental systems

n/a	Involved in the study	
☐	☒	Antibodies
☒	☐	Eukaryotic cell lines
☒	☐	Palaeontology
☐	☒	Animals and other organisms
☒	☐	Human research participants
☒	☐	Clinical data

Methods

n/a	Involved in the study	
☒	☐	ChIP-seq
☐	☒	Flow cytometry
☒	☐	MRI-based neuroimaging

Antibodies

Antibodies used	List of antibodies used, details of clone and company in Supplemental Information (Table 2)
Validation	Commercial antibodies used in this study target validated by manufacturers (and quality control), please see websites below for relevant materials and references of previous use:<br>BD - https://www.bdbiosciences.com/en-us<br>eBioscience/Invitrogen - https://www.thermofisher.com/uk/en/home/life-science/antibodies.html<br>Biolegend - https://www.biolegend.com

Animals and other organisms

Policy information about studies involving animals; ARRIVE guidelines recommended for reporting animal research

Laboratory animals	Female BALB/c mice ages 10-14 weeks; 22-25g<br>Female Lewis rats age 14 weeks; ~200g
Wild animals	Study did not involve wild animals
Field-collected samples	Study did not involve field-collected samples
Ethics oversight	Mouse and rat studies were carried out under the United Kingdom's Animals (Scientific Procedures) Act of 1986 and local ethical approval from Imperial College London. The rat posterolateral lumbar fusion procedure was performed in accordance with an approved protocol reviewed and administered by Beaumont Health's IACUC, which conformed with applicable federal and local regulations.

Note that full information on the approval of the study protocol must also be provided in the manuscript.

Flow Cytometry

Plots

Confirm that:

☒ The axis labels state the marker and fluorochrome used (e.g. CD4-FITC).

☒ The axis scales are clearly visible. Include numbers along axes only for bottom left plot of group (a 'group' is an analysis of identical markers).

☒ All plots are contour plots with outliers or pseudocolor plots.

☐ A numerical value for number of cells or percentage (with statistics) is provided.

Methodology

Sample preparation	Blood CFU-Fs had been expanded in culture for a maximum of three to four passages. Cells were trypsinised (0.05% trypsin EDTA), collected as a single cell suspension after neutralisation of trypsin, washed and resuspended in FACS buffer (PBS + 3% FBS). Cells were blocked with 10% mouse serum for 30mins (on ice) before they were stained with fluorochrome-conjugated monoclonal antibodies (30mins on ice; FACS buffer 10% mouse serum), washed twice and fixed using BD fix overnight in the fridge.<br>For freshly isolated cells from blood: Blood was RBC-lysed using ACK lysis buffer for 4 mins on ice, washed twice with DMEM +20% FBS. A second round of RBC-lysis was carried out as described. Cells were resuspended in D20 and counted. Cells were then resuspended in FACS buffer and the same staining protocol was carried out as described above.
Instrument	BD Fortessa with BD FACS Diva software
Software	BD FACS Diva software (collection) and FlowJo v7 and v10 (analysis)
Cell population abundance	For % PaS (out of total nucleated cells in blood): 0.25% in AMD3100 group and 0.47% in AMD3100/B3AR agon' group.
Gating strategy	With regards to cultured cells FSC/SSC-A gating to select cell population (exclude debris), then FSC-A/FSC-H and SSC-A/SSC-H to select single cells (exclude any doublets). Isotype controls and Fluorescence minus one (FMO) controls were used for single stain comparison and to define boundaries.<br>With regards to freshly isolated cells FSC/SSC-A gating to select cell population (exclude debris), then FSC-A/FSC-H and SSC-A/SSC-H to select single cells (exclude any doublets). Fluorescence minus one (FMO) controls were used to define boundaries and gate positive populations.

☒ Tick this box to confirm that a figure exemplifying the gating strategy is provided in the Supplementary Information.
